# Personalized medicine approach confirms a milder case of ABAT deficiency

**DOI:** 10.1186/s13041-016-0273-8

**Published:** 2016-12-01

**Authors:** A. Besse, A. K. Petersen, J. V. Hunter, V. Appadurai, S. R. Lalani, P. E. Bonnen

**Affiliations:** 1 Department of Molecular and Human Genetics, Baylor College of Medicine, Houston, TX USA; 2 Department of Radiology, Baylor College of Medicine and Texas Children’s Hospital, Houston, TX USA

## Abstract

ABAT deficiency (OMIM 613163) is a rare inborn error of metabolism caused by recessive variants in the gene 4-aminobutyric acid transaminase (ABAT), which is responsible for both the catalysis of GABA and maintenance of nucleoside pools in the mitochondria. To date, only a few patients have been reported worldwide. Their clinical presentation has been remarkably consistent with primary features of severe psychomotor retardation, encephalopathy, hypotonia, and infantile-onset refractory epilepsy. We report a new case of ABAT deficiency that marks an important departure from previous clinical findings. The patient presented at age 6 months with global developmental delay, hypotonia, hypersomnolence and mild choreiform movements. At age 18 months, the subject’s clinical presentation was still milder than all previously reported patients and, most notably, did not include seizures. Clinical whole exome sequencing revealed two heterozygous *ABAT* missense variants that are rare and predicted damaging, but never before reported in a patient and were reported as variants of unknown significance. To test the potential pathogenicity of the variants identified in this patient we developed a cell-based system to test both functions of the ABAT protein via GABA transaminase enzyme activity and mtDNA copy number assays. This systematic approach was validated using vigabatrin, the irreversible inhibitor of ABAT, and leveraged to test the functionality of all ABAT variants in previously reported patients plus the variants in this new case. This work confirmed the novel variants compromised ABAT function to similar levels as variants in previously characterized cases with more severe clinical presentation, thereby confirming the molecular diagnosis of this patient. Additionally, functional studies conducted in cells from both mild and severe patient fibroblasts showed similar levels of compromise in mitochondrial membrane potential, respiratory capacity, ATP production and mtDNA depletion. These results illustrate how cell-based functional studies can aid in the diagnosis of a rare, neurological disorder. Importantly, this patient marks an expansion in the clinical phenotype for ABAT deficiency to a milder presentation that is more commonly seen in pediatric genetics and neurology clinics.

## Introduction

ABAT deficiency (OMIM 613163), also called GABA-Transaminase deficiency, is a rare inborn error of metabolism caused by recessive variants in the gene 4-aminobutyric acid transaminase (ABAT). To date, only a few patients have been reported worldwide [1–3]. Their clinical presentation has been remarkably consistent with primary features of severe psychomotor retardation, hypotonia, encephalopathy, and infantile-onset refractory epilepsy.

ABAT encodes for GABA transaminase, the enzyme responsible for catabolizing the principal inhibitory neurotransmitter gamma-aminobutyric acid (GABA). Recent work demonstrated that ABAT has a second role in the mitochondrial nucleoside salvage pathway converting dNDPs to dNTPs [1]. This function of the protein is essential for maintenance of nucleoside pools available to the mitochondrial genome replication machinery. Thus inhibition of this protein results in both elevated levels of GABA and reduced copy number of mitochondrial genome in patient tissues [1].

Current diagnostic paradigms for genetic disorders include exome sequencing. However, typically 25% of cases receive a molecular diagnosis [4, 5]. The standards and guidelines outlined by American College of Medical Genetics and Genomics and the Association of Molecular Pathology for interpretation of sequence variants point to functional validation of missense variants as essential for proof of pathogenicity of a variant of unknown significance [6]. These should be ‘well-established in vitro or in vivo functional studies supportive of a damaging effect on the gene or gene product’. For most genes known to cause single gene Mendelian disorders such assays have not been established in the clinical diagnostic setting despite the rising need driven by increased use of exome sequencing in this setting.

We describe a new case of ABAT deficiency with a milder clinical presentation than previous patients. We review previous cases and discuss the common clinical hallmarks clinical of this disorder. Additionally, we devised a non-invasive strategy for functionally vetting variants in the ABAT gene for potential pathogenicity, demonstrated its efficacy, and leveraged this platform for providing functional validation of pathogenicity of variants and substantiate the molecular diagnosis for this unusual patient. Importantly, this patient marks an expansion in the clinical phenotype for ABAT deficiency to a milder presentation that is more commonly seen in pediatric genetics and neurology clinics and our results illustrate a non-invasive approach that can aid in the diagnosis of a rare, neurological disorder.

## Methods

### Human subjects

All subjects and their families were consented to an Institutional Review Board -approved protocol for participation in research study. Genomic DNA was extracted from blood and fibroblasts according to standard protocols. Genetic testing including exome sequencing was conducted at Baylor Genetics Laboratories, Houston, Texas. All variants identified were annotated using HG19, NM_000663.4, and NP_000654.2.

### Cell culture

Glioblastoma cells T98G were obtained from the American Type Culture Collection (Manassas, VA). The cells were grown in complete Eagle’s Minimum Essential Medium (MEM) supplemented with 10% FBS. Separately, T98G cells were cultered with MEM and various doses of vigabatrin (Sigma-Aldrich, St. Louis, MO). The growth medium with vigabatrin was replaced every day for 10 days. After 10 days, the cells were harvested to assess mtDNA content and GABA-T enzymatic activity. Fibroblasts were grown in MEM supplemented with 15% FBS, 1x MEM-vitamins, 2 mM L-Glutamine, 1 mM sodium pyruvate, 1x penicillin/streptomycin, 1x non-essential amino acids, and 0.41 μM uridine. Separately, in order to synchronize cells in G0, cells were grown in in “low-serum” media 1% FBS for 10 days. Cells were maintained as a monolayer in a humidified 37C, 5% CO2 atmosphere.

### Quantitation of mtDNA copy number

mtDNA content of the control and patients cells were analyzed using real-time qPCR method as previously described [1]. The ND1 region of human mtDNA was amplified using forward primer 5′ GTCAACCTCGCTTCCCCACCCT 3′ and reverse primer 5′ TCCTGCGAATAGGCTTCCGGCT′, giving a fragment of 108 bp; A fragment of human beta 2 microglobulin gene was amplified using forward primer 5′ CGACGGGAGGGTCGGGACAA 3′ and reverse primer 5′ GCCCCGCGAAAGAGCGGAAG 3′ giving a fragment of 118 bp. The real time PCR reaction was performed in triplicate for each DNA sample using a StepOne Plus RT-PCR system (Applied Biosystems). PerfeCTa SYBR Green FastMix ROX was purchased from Quanta Biosciences. 500nM of each primer, and 10 ng of total genomic DNA extract were used per reaction. After real-time PCR amplification, the mtDNA content (mtDNA/B2M ratio) was calculated using the formula: mtDNA content =1/2^ΔCt^, where ΔCt = Ct^mtDNA^ − Ct^B2M^.

### GABA-T enzymatic activity assay

GABA-T enzymatic activity was determined using a GABA-T assay kit (Biomedical Research Service Center, Buffalo University, NY) according to manufacturer instructions. Briefly, the assay is based on sequential GABA transamination reaction and glutamate dehydrogenase reaction, which couples the reduction of iodonitrotetrazolium to iodonitrotetrazolium-formazan (ε = 18 mM^-1^cm^-1^ at 492 nm). Reactions were terminated by adding 3% acetic acid (Sigma-Aldrich, St. Louis, MO) and optical density (OD) at 492 nm was measured using a plate reader (Phoenix Sunrise, Tecan). Readings were averaged and control wells readings were subtracted from sample wells readings (ΔOD). GABA-T activity was calculated using the following formula: GABAT activity (μmol/(L.min) = (ΔOD x 1000 x 155 μl)/(60 min x 0.6 cm x Ɛ x 10 μl) = ΔOD x 23.92, where 155 μl is the total reaction volume, 0.6 cm the light path in the 96 well plate, 10 μl the volume of sample in each well.

### shRNA knock down of ABAT

Oligonucleotides containing shRNA hairpins that target the 3’UTR of ABAT were custom designed and cloned into the pGIPZ vector (GE Healthcare). Control fibroblasts were infected with two independent shRNA hairpins plus a scrambled shRNA and 72 hours post infection QRT-PCR and Western Blot for ABAT were performed on these cells as described.

### Quantitation of ABAT mRNA by qRT-PCR

RNA was extracted and cDNA synthesized using standard protocols. Quantitative real-time PCR experiments were performed using a StepOne Plus RT-PCR system (Applied Biosystems), RT2 qPCR Primer Assays for Human GAPDH and ABAT (SABiosciences) and PerfeCTa SYBR Green FastMix ROX (Quanta Biosciences). All assays were conducted in triplicate and the results are shown as the average and standard deviation.

### Cloning and functionally testing ABAT variants

To assess the effect of ABAT mutations on mtDNA content and GABA-T enzymatic activity, we expressed ABAT patient variants in glioblastoma T98G cells using a lentiviral delivery system as previously described [1]. Wild-type ABAT entry ORF clone (NM_000663.3; clone ID: IOH13638) was utilized to generate ABAT ORFs containing patient variants by site-directed mutagenesis as described [1]. The following mutant PCR primers were used:

(c.631C > T):ctgccccgactacagcatcTtctccttcatgggcgcgttccatgggagga; (c.275G > A):gttgatgtggacggcaaccAaatgctggatctttattcccagatctcctc); (c.1433 T > C):attcgtttccgtcccacgcCggtcttcagggatcaccacgctcacctgtt; (c.659G > A):atgggcgcgttccatgggaAgaccatgggttgcttagcgaccacgcactc; (c.454C > T): ccttgctctcggtggctTccaaagggatgtc; (c.1393G > C): aaggtgtggtgttgCgtggctgtggtgac

Mutation p.Q66del was generated by PCR using ABAT-specific primers fused to attB1/B2 recombination sequences (Invitrogen) designed following manufacture’s recommendations.

shRNA hairpins that target the ABAT 3′UTR and a scrambled shRNA were transferred from the pGIPZ vector into the pGIPZ-GW by enzymatic digestion (XhoI + MluI) and ligation. Then the cDNAs corresponding to mCherry, ABAT wild-type, or ABAT mutants were transferred from Donor223 vector (Invitrogen) into the pGIPZ-GW by LR recombination reaction (Invitrogen).

Virus production was conducted as previously described [7]. Infectious lentiviral supernatant was used to infect healthy growing fibroblasts with polybrene (Sigma) before selection by puromycin. After puromycin selection, the cells were maintained in culture for 10 days and then harvested. mtDNA content and GABA-T activity were determined as described.

### Mitochondrial membrane potential

Mitochondrial membrane potential was assessed using MitoProbe DilC1(5) Assay kit for Flow Cytometry (Life Technologies) as described [7]. Briefly, confluent cells were harvested, washed in pre-warmed 37 °C 1X PBS and counted using Vi-CELL cell viability analyzer (Beckman Coulter). The cells were either stained directly with the DilC1(5) dye at a final concentration of 50 nM for 30 minutes at 37 °C, 5% CO2 or pre-incubated with 50 mM of carbonyl cyanide 3-chlorophenylhydrazone (CCCP) for 15 minutes at 37 °C, 5% CO2 prior to staining with DilC1(5). After staining, the cells were washed and visualized by flow cytometry, and the data analyzed with FlowJo software. Triplicate results are reported and are shown as the average and standard deviation.

### Respiration studies using micro-scale oxygraphy

XF24 extracellular flux analyzer from Seahorse Biosciences was used to measure oxygen consumption rates following standard protocols. Cells were plated the previous day of experiment at a density of 30,000 cells per well. XF assay medium (5 mM glucose/galactose, 2 mM Pyruvate) in XF base media (Seahorse Biosciences) was prepared and pH adjusted to 7.0. Oxygen consumption rates were measured for 3 min with 3 min of mixing and 2 min of waiting period in between addition of each cellular stress reagents: 500nM Oligomycin, 500nM FCCP, 100nM Antimycin and 100nM Rotenone (Sigma-Aldrich). Cells were counted post-analysis using ViCell cell viability analyzer (Beckman Coulter) and counts were used to normalize OCR rates. Each sample was analyzed in 5 replicate wells.

## Results

### Clinical description

Subject 1 was a male of European ancestry who came to clinical attention at 6 months of age with the non-specific findings of global hypotonia, developmental delay and mild choreiform movements. In addition, the child displayed hypersomnolence to the extent of sleeping 20 – 22 hours per day. No consanguinity or family history of neurological disorder was reported.

Cranial MRI conducted on Subject 1 at 9 months of age showed extensive T2 hyperintensity in the deep white matter that is evidence of diffuse, mild deep white matter hypomyelination. The pattern of myelination observed is consistent with the expected pattern of myelin maturation at 4 months of life, indicating a lag of 5 months behind the subject’s chronological age. There were no structural brain abnormalities.

An EEG conducted at age 13 months showed background activity was diffusely slow with a slow occipital rhythm, indicating the presence of a diffuse disturbance in cerebral function. There were no focal or lateralizing features and no epileptiform activity was recorded. A subsequent EEG at 16 months continued to display diffuse slowing of background activity with no seizures recorded even though multiple choreiform movements and staring spells occurred during recording. However, this study did observe the presence of spike and wave complexes in the right central, left parietal, and midline centroparietal regions that increased in frequency during sleep. These findings indicated the presence of focal, potentially epileptogenic processes in these regions.

At the age of 18 months the patient had severe hypotonia, developmental delay without regression and oculomotor apraxia. The subject displayed some fine choreiform movements but to that point no clinical seizures. He had reached some developmental milestones: he had some head control when sitting, tracked faces and smiled appropriately, ate and drank by mouth including solid food.

### Genetic and biochemical investigations reveal ABAT deficiency

Diagnostic whole exome sequencing reported two variants of unknown significance in *ABAT*. The subject appeared compound heterozygous for two missense variants: *ABAT* NM_000663.4 c.454C > T;p.Pro152Ser and c.1393G > C; p.Gly465Arg. Sanger sequencing confirmed segregation in the family consistent with an autosomal recessive disorder, with c.454C > T present in the mother and c.1393G > C in the father. Both variants scored maximally damaging by SIFT, PolyPhen-2, LRT, MutationTaster. Indicators of evolutionary constraint were high for both variants with Gerp and PhyloP 5.6 and 2.7 for p.Pro152Ser and 5.1 and 2.4 p.Gly465Arg. The composite metric CAAD Phred also scored high with of 30 and 26. Neither variant was observed in ExAC, Exome Sequencing Project or 1000 genomes project. Neither variant has been previously reported in an ABAT deficiency patient and since both were missense variants, by the guidelines of American College of Medical Genetics these are considered variants of uncertain significance (VUS) and in the diagnostic exome report were classified as VUS.

Cerebral spinal fluid (CSF) displayed GABA free level of 247 nmol/L (17-67) and total level of 33.4 umol/L (4.2-13.4). CSF was tested for neurotransmitters prior to exome sequencing and was normal, but GABA was not included in this panel of neurotransmitters. Subject 1 exhibited normal plasma and CSF amino acids.

### Phenotype expansion in the clinical presentation of ABAT deficiency

The clinical presentation of Subject 1 overlaps with previously reported cases of ABAT deficiency while extending the phenotype to a milder presentation (Table 1). All cases reported show profound global hypotonia and hypersomnolence. All cases were demonstrated to have elevated GABA in CSF or brain. However, significant distinctions can be made between this newest case of ABAT deficiency with regard to developmental milestones, seizure activity and cranial MRI abnormalities.

**Table 1:** Clinical Features of subjects with ABAT Deficiency

		Family 1		Family 2		Family 3		Family 4
		Subject 1	Subject 2	Subject 3	Subject 4	Subject 5	Subject 6	Subject 7
	*ABAT* Variants	c.454C>T, p.Pro152Ser ; c.1393G>C, p.Gly465Arg	c.631C>T, p.Leu211Phe ; c.631C>T, p.Leu211Phe			c.659G>A,p.Arg220Lys ; c.1433T>C, p.Leu478Pro		c.275G>A, p.Arg92Gln ; c.199-?_316+?del, p.Asn67ValfsTer8
	Gender	M	F	M	F	F	M	F
	Age at last follow-up	2 yr	7 yr	5 yr	1 yr	2	12 months	7 yr
	Current age	2 yr	deceased	7 yr	deceased	deceased	deceased	-
	Age of clinical presentation	6 mo	3 mo	2 mo	2 mo	birth	birth	7 mo
	Age at death	-	7 yr	-	1 yr	2 yr	12 mo	-
Ophthalmologic	Cortical visual impairment	No	Yes	Yes	Yes	NA	NA	NA
	Optic nerve atrophy	No	Yes	Yes	Yes	NA	NA	NA
Developmental Milestones	Global developmental delays	Yes	Severe	Severe	Severe	Severe	Severe	Severe
	Hold up head	2 years	No	No	No	No	No	No
	Roll over	9 mo	No	No	No	No	No	No
	Sit up	12 mo (c assistance)	No	No	No	No	No	No
	Ambulation	No	No	No	No	No	No	No
	Expressive language	coos/babbles	No	No	No	No	No	No
Neurologic	Hypotonia	Yes	Yes	Yes	Yes	Yes	Yes	Yes
	Abnormal movements	Yes	No	No	No	No	No	No
	Hypersomnia	Yes	Yes	Yes	Yes	Yes	Yes	Yes
	Seizures, age of onset	No	3 mo	4 mo	4 mo	birth	birth	7 mo
	Seizures, controlled by medication	-	No	No	No	No	No	No
	EEG	multi-focal spikes, diffuse slowing	Hypsarrhythmia at 3 mo	Normal at 3 mo, Hypsarrhythmia at 6 mo	Normal at 3 mo, Hypsarrhythmia at 6 mo	multi-focal spikes, diffuse slowing	multi-focal spikes, diffuse slowing	spike-wave, diffuse slowing
Cranial MRI	Atrophy	No	Yes	Yes	Yes	-	-	Yes
	Decreased white matter	No	Yes	Yes	Yes	-	-	Yes
	Hypomyelination	Yes	Yes	Yes	Yes	-	-	Yes
Metabolic	GABA levels	Elevated in CSF	Elevated by brain MRS	Elevated by brain MRS	Elevated by brain MRS	Elevated in CSF	-	Elevated by brain MRS

Family 1 this manuscript

Family 2 Besse et al 2015 [1]

Family 3 Jaeken et al 1984 [3]

Family 4 Tsuji et al 2010 [2]

The clinical presentation of all previously reported cases of ABAT deficiency is summarized in Table 1 [1–3]. In addition, Subject 4, a sibling of Subjects 2 and 3, is newly reported here and can be briefly described as having a highly similar clinical presentation to her siblings originally described in Besse *et al*, 2015. All cases except Subject 1 showed profound developmental delay with no patients reported to reach any significant developmental milestones. In contrast, Subject 1 achieved some milestones, albeit delayed, including rolling (9 months), sitting with assistance (12 months) and eating solid food (18 months).

Seizure activity reported in previous patients was neonatal- or infantile-onset. Subjects 2,3,4 displayed hypsarrhythmia starting at ages 3-6 months that did not respond to treatment [1]. Subjects 5,6 exhibited convulsions in the first day of life [3]. Subject 7 first experienced seizures at 8 months [2]. All of these patients’ seizures persisted throughout life and none were controlled by medications. In contrast, at 18 months Subject 1 had not yet displayed overt seizures.

**Fig. 1:**
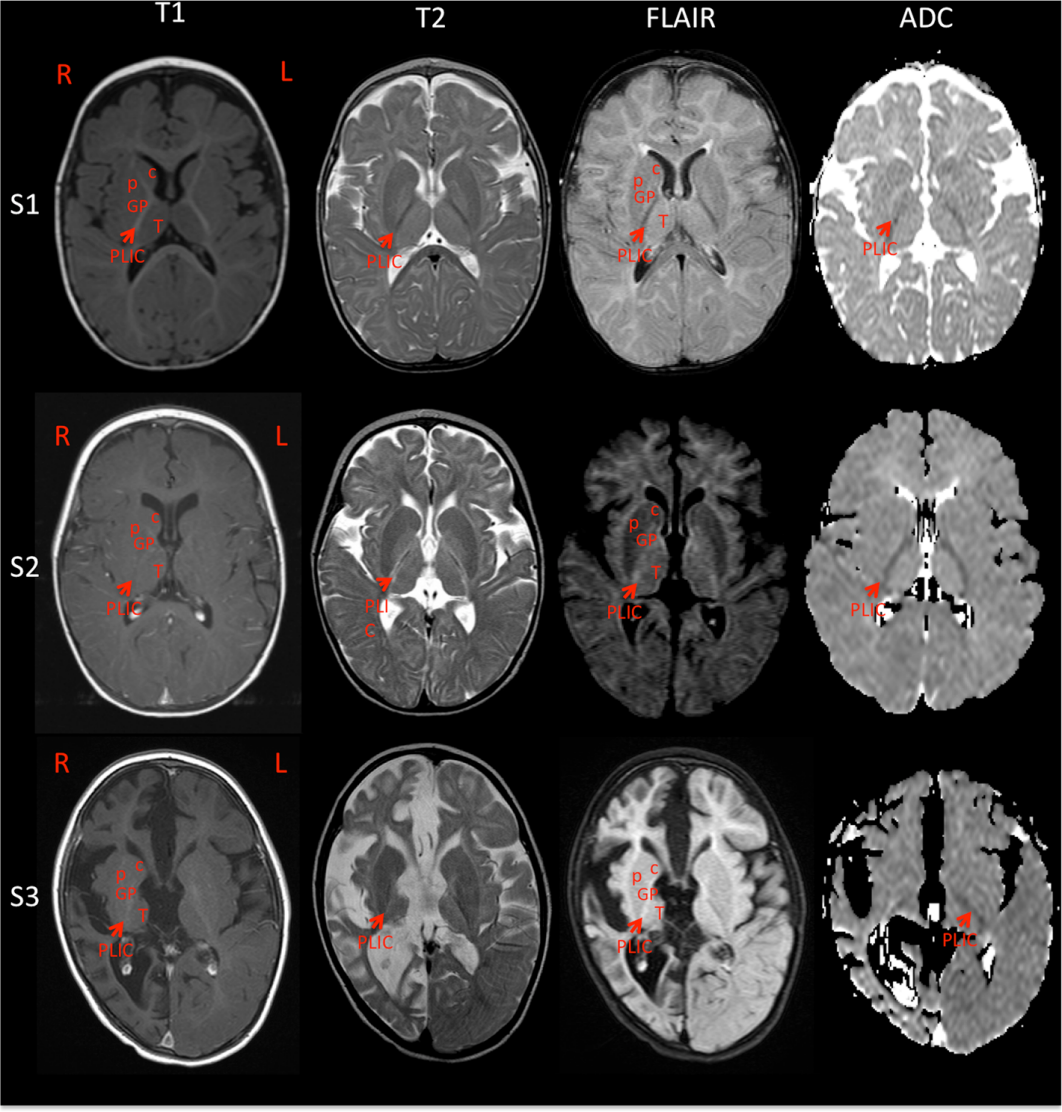
Cranial MRI shows a consistent pattern of hypomyelination across ABAT patients. MRI of Subject 1 (S1) at age 9 months shows extensive T2 hyperintensity in the deep white matter that lags ~5 months behind the patient’s chronological age, suggesting diffuse mild hypomyelination. However, the anterior limb of the internal capsule is hyperintense on T1, and partially hypointense on T2, with a non-restricted ADC map. In contrast, while Subjects 2 (S2) and 3 (S3) are older at 13 and 17 months, myelination in the posterior limbs of the internal capsules is less complete and associated with restricted diffusion on ADC, suggestive of more severe hypomyelination. Additionally, Subjects 2 and 3 exhibit diffuse yet mild cerebral atrophy. Subject 3 also exhibits asymmetry with disproportionate right sided atrophy. C: caudate, P: putamen, GP: globus palidus, T: thalamus, PLIC: posterior limb of the internal capsule

Subject 1 showed clear delays in myelination like other reported subjects with ABAT deficiency, however, the imaging phenotype of Subject 1 is notably milder than other patients. Illustrated in Fig. 1, the cranial MRI for Subjects 2 and 3 demonstrated that while Subjects 2 and 3 were older at time of imaging (13 and 17 months respectively), myelination in the posterior limbs of the internal capsules (PLICs) is even less complete than Subject 1 at age 9 months, and is associated with restricted diffusion on ADC, suggestive of more severe hypomyelination. Also, in contrast to Subject 1, these two subjects exhibited diffuse yet mild cerebral atrophy. In the 13 month old, bilateral hypomyelination was also visualized in the brainstem middle cerebral peduncle, central tegmental areas, and medial thalami (not pictured). The 17 month old also exhibited asymmetry with disproportionate right-sided atrophy. Similarly, imaging in Subject 7 showed marked hypomyelination, with diffusion weighted images revealing high signal intensity in the internal and external capsules and much of the subcortical white matter, with restricted diffusion abnormality [2]. These imaging observations between subjects correlated with their clinical severity.

### Non-invasive functional testing for ABAT deficiency

**Fig. 2:**
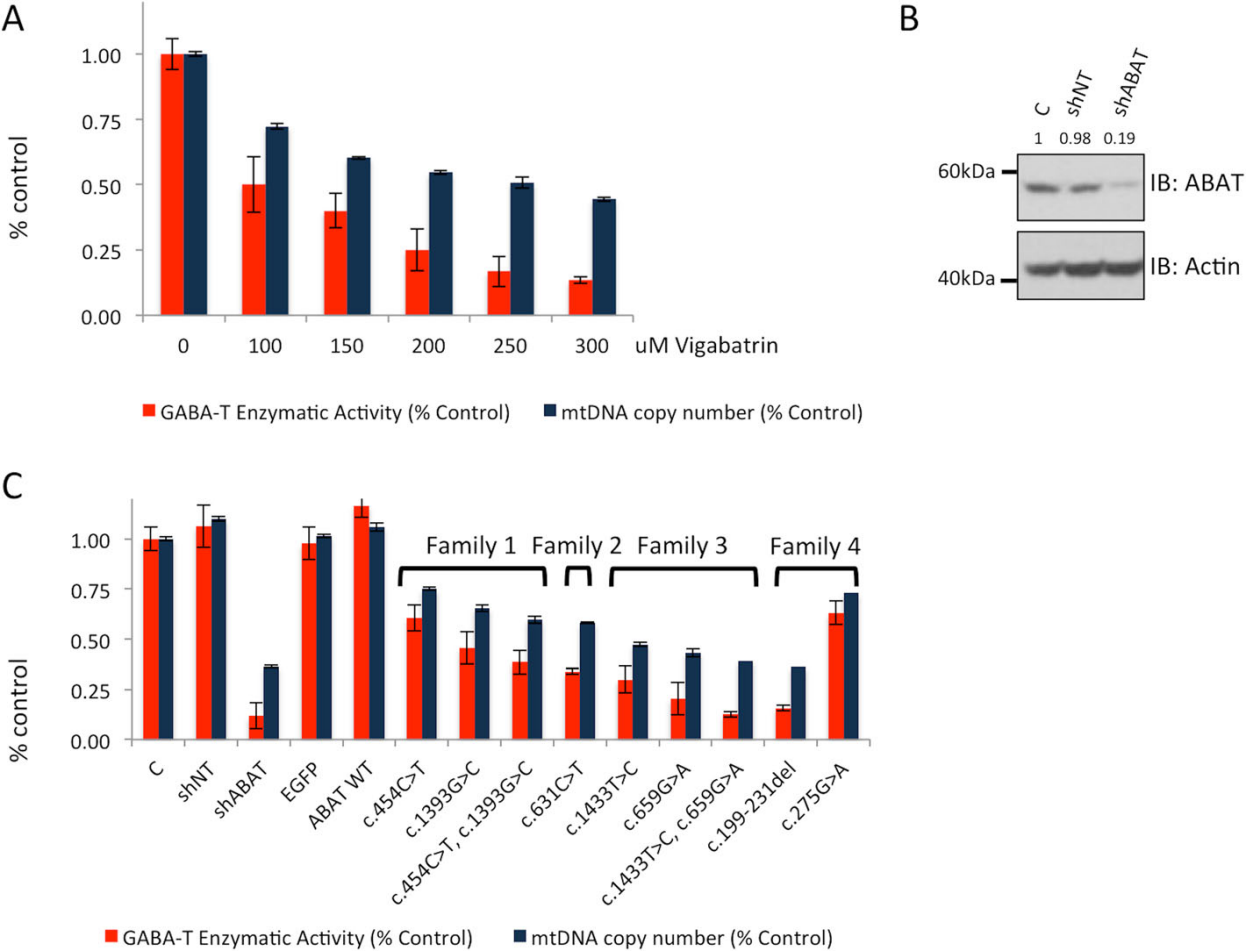
GABA-T enzymatic activity and mtDNA copy number are diminished in cells expressing ABAT deficiency patient variants. **a**. Cells cultured in the presence of vigabatrin, the irreversible inhibitor of ABAT, show deficits in GABA-T activity and mtDNA copy number that follow a dose-response. **b**. Immunostaining for ABAT protein levels in cells transformed with non-targeting shRNA (shNT) and shRNA targeting ABAT (shABAT) show ABAT protein levels 19% of control in cells transformed with shABAT. **c**. GABA-T enzymatic activity and mtDNA copy number was measrured in cells transformed with shNT, shABAT, eGFP, open reading frame for wild-type ABAT (ABAT WT), and the mutations identified in ABAT deficiency patients. All experiments conducted in T98G cells

ABAT functions in the mitochondrial inner membrane to catabolize GABA and to produce nucleoside pools essential for the maintenance of the mitochondrial genome. We tested both functions of the protein in a human cellular model. Vigabatrin is an irreversible inhibitor of ABAT, typically prescribed for its anticonvulsant effects resulting from its ability to elevate GABAergic tone. In initial experiments, control cells were cultured in the presence of vigabatrin at varying doses ranging from 0 – 300 uM. mtDNA content decreased as dose increased and was diminished to 50% of control at the 200 uM dose which we have previously observed [1] (Fig. 2a). The same cells under same conditions were analyzed for GABA-T activity to test this function of ABAT. Like mtDNA content, GABA-T activity diminished with increasing dose of Vigabatrin and uniformly showed greater compromise compared to control than mtDNA content at every dose. GABA-T activity was 50% of control activity at the 100 uM dose and 25% of control at 200 uM in this cellular model.

We next utilized this cellular model to test the functional consequences of *ABAT* variants identified in all reported patients for both functions of ABAT. In this allelic series experiment we utilized genetic knockdown and expression of wildtype ABAT as controls. The shRNA-induced knock-down of ABAT expression reduced ABAT protein levels to 19% of control (Fig. 2b) and these cells displayed GABA-T activity at 14% of control and mtDNA copy number at 33% of control (Fig. 2c). Non-transfected controls cells and those transfected with non-targeting hairpin, eGFP, or wildtype ABAT in cells all resulted in GABA-T activity and mtDNA content at highly similar ‘normal’ levels (Fig. 2c). Every patient allele tested universally resulted in compromised function in both GABA-T activity and mtDNA content. mtDNA content in cells expressing individual patient alleles ranged from 36 – 75% of control. Similarly, GABA-T activity was decreased and ranged from 15 – 63% of control.

The allele present in Family 2, c.631C > T, reduced GABA-T activity to 31% and mtDNA to 51%. Given that the affected individuals from this family are homozygous for this allele this may represent a reasonable proxy for activity in patient tissue. Subjects with ABAT deficiency in Family 3 were compound heterozygous for ABAT c.199-231del and c.275G > A and each of these alleles individually compromised activity to 15% (GABA-T) / 36% (mtDNA) and 63% (GABA-T) / 73% (mtDNA) respectively. Given the null allele present in this family we presume the ABAT homodimer containing both of these alleles would function more closely to the activity of the null allele than the missense allele.

In an effort to phenocopy compound heterozygous patient tissue as closely as possible in this cellular system we created double mutant knock-in alleles. Affected individuals in Family 4 are compound heterozygous for c.1433 T > C and c.659G > A. Individually these alleles diminished activity to 27% (GABA-T) / 47% (mtDNA) and 20% (GABA-T) / 43% (mtDNA) respectively, and the double mutant bearing both alleles showed further compromise with 16% (GABA-T) / 39% (mtDNA) which is essentially the same level of activity as the shRNA knock-down and null allele (c.199-231del). Subject 1 is compound heterozygous for c.454C > T and c.1393G > C. These two alleles individually and combined demonstrate less compromise in activity compared to others. Allele c.454C > T shows 61% (GABA-T) / 75% (mtDNA) which is comparable to the performance of the Subject 3’s milder allele which is present in this patient in combination with a null allele. The second allele in Subject 1 is also a milder allele showing 46% (GABA-T) / 65% (mtDNA) activity. The double mutant shows 39% (GABA-T) / 60% (mtDNA), which is comparable to deficiency in activity observed in the cells cultured with 150 uM Vigabatrin.

### Cellular investigations demonstrate similarly compromised mitochondrial bioenergetics in mild and severe ABAT deficiency cases

**Fig. 3:**
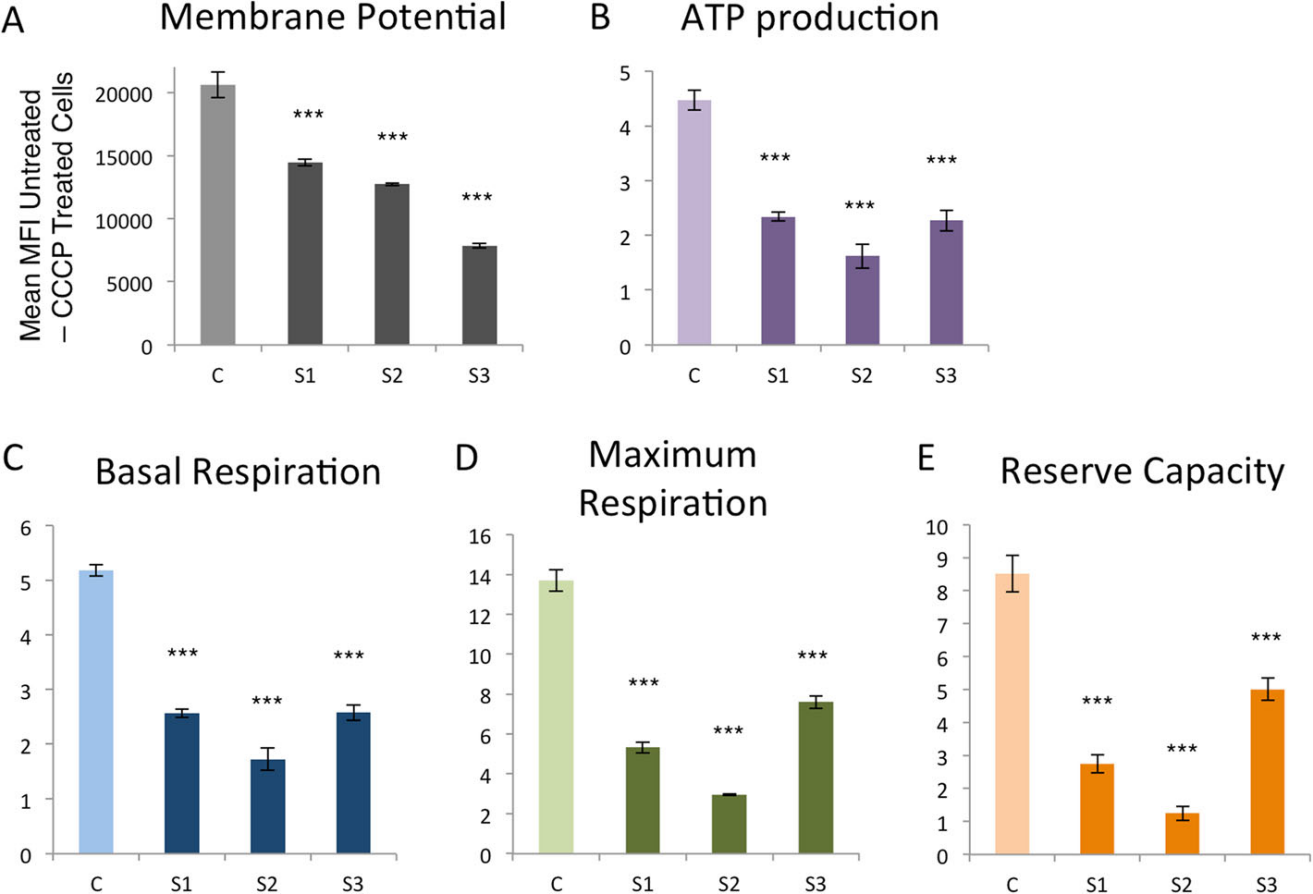
Mitochondrial bioenergetics are compromised in ABAT deficiency patients. **a**. Mitochondrial membrane potential is reduced in fibroblasts from ABAT deficiency patients Subject 1 (S1), Subject2 (S2), and Subject3 (S3). **b**. Microscale oxygraphy demonstrated that ATP production, **c**. Basal respiration, **d**. Maximum respiration, and **e**. Reserve capacity were all relatively similarly compromised in ABAT deficiency patients, both mild (S1) and severe (S2, S3)

We examined the inner mitochondrial membrane potential in a fibroblast cell line established from Subject 1 and compared this to membrane potential in fibroblasts from two previously reported affected siblings with ABAT deficiency, Subjects 2 and 3. The electron transfer activity of the respiratory chain complexes pumps protons from the mitochondrial matrix into the intermembrane space, creating an electrical potential across the inner mitochondrial membrane. This membrane potential was significantly compromised in all three subjects compared to controls (Fig. 3a). Subject 1 is the mildest and least compromised, however there is more differential between the two affected siblings, Subjects 2&3, than between Subject 1 and Subjects 2&3 who carry the same mutation and we cannot conclude that Subject 1 fibroblast has a milder phenotype, and rather must attribute these differences to variation amongst immortalized cell lines.

Oxygraphic measurements of respiration in live cells demonstrated reduced capacity in basal and maximum respiration as well as reserve capacity (Fig. 3b-e). ATP production was also compromised to approximately 50%. All three patient cell lines showed similar levels of compromise.

### Decreased mtDNA copy number in patient cells

**Fig. 4:**
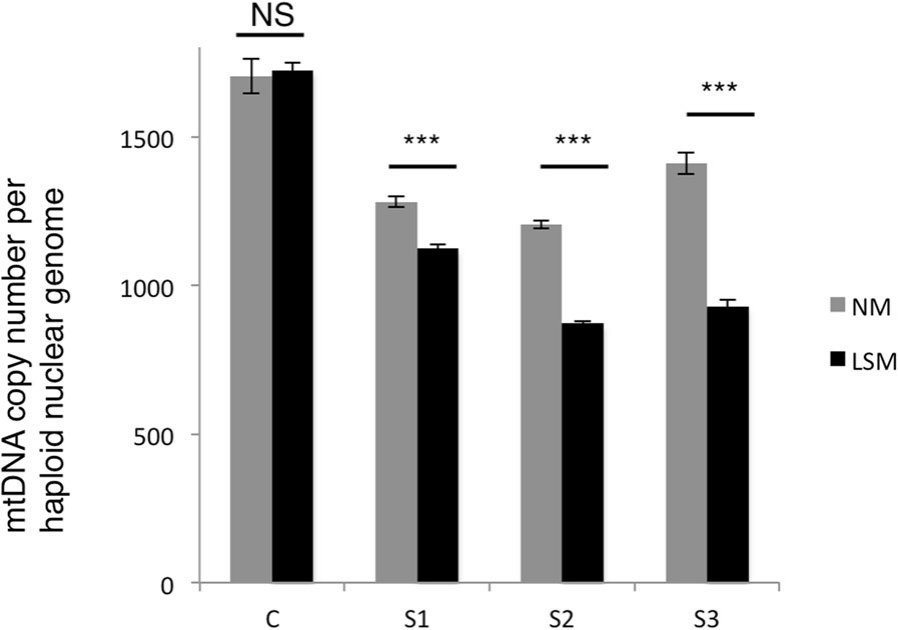
mtDNA copy number is decreased in ABAT deficiency patients. Healthy control (C) and patient fibroblasts (S1, S2, S3) were either grown in media with FBS 15% (NM) or in “low-serum” media FBS 1% for 10 days (LSM) mtDNA copy number was measured. NS = non-statistically significant, *** *p*-value < 0.0001

ABAT deficiency has been demonstrated to result in decreased mtDNA content in patient cells [1]. In normal media where cells are cycling, the reduction in mtDNA copy number is approximately 70 - 80% in all three patient cell lines (Fig. 4). Low serum media pauses cell cycle in G0 which forces mitochondria to rely on the mitochondrial nucleoside salvage pathway to supply nucleosides to the mtDNA replication machinery, which continues even in G0 while the nuclear genome replication machinery is halted. ABAT was shown to be essential for converting dNDPs to dNTPs in the mitochondrial nucleoside salvage pathway and as a result when ABAT deficiency patient cells are cultured in low serum media they show greater reduction in mtDNA content. Subject 1 shows a lesser decrease in mtDNA content when in low serum media compared to Subjects 2 and 3. Subject 1 shows 65% of control while Subjects 2 and 3 display 55%, a statistically significant difference p = 0.0004 (Fig. 4).

## Discussion

We describe a subject who represents a phenotypic expansion for ABAT deficiency. We devised and validated a non-invasive strategy for functionally vetting variants in *ABAT* and successfully provided functional validation of pathogenicity of this subject’s VUS. Further, we compared the cellular function of this patient’s fibroblasts to previous more severely affected cases and show similar levels of dysfunction in mitochondrial membrane potential, respiratory capacity, ATP production, and copy number of the mtDNA genome.

Review of published cases of ABAT deficiency converges upon signature clinical features across cases. All cases reported showed severe encephalopathy, profound global hypotonia, elevated GABA in CSF or brain, cranial MRI findings of hypomyelination with restricted diffusion abnormality in PLICs. Equally important to note are the differences between clinical presentations. In particular, this patient demonstrates that a lack of overt seizures and, notably, the ability to obtain some developmental milestones, albeit delayed. Additionally, cranial MRI for subjects with more severe clinical presentation demonstrated varying degrees of atrophy in addition to more severe delays in myelination with corresponding areas of restricted diffusion in the PLICs.

The cellular system innovated and leveraged to test the functional consequences of *ABAT* variants identified in all reported patients for both functions of ABAT is a model that can be extrapolated for other genes. The ABAT deficiency patient allelic series showed that all known patient mutations inhibit GABA-T activity to less than 30% of normal activity, except for three. One of these mutations was found as a compound heterozygous with a null mutation and the patient had a severe phenotype. Two other mutations that had less severe effects *in vitro* were observed in the milder patient, Subject 1, lending some support for the ability of this *in vitro* assay to distinguish severity of mutation and support for the notion that this milder patient’s phenotype may be milder due to having two less debilitating mutations.

Notably, both vigabatrin and patient mutations appear to compromise GABA-T activity more so than mtDNA copy number. The 200 uM dose of vigabatrin reduced GABA-T to 25% of normal and mtDNA to 50% of normal. This dose of vigabatrin is comparable to what is prescribed to control seizures and impairs GABA-T activity and mtDNA copy number to similar levels as the more severe patient mutations; and yet have very different outcomes in ABAT deficiency patients versus patients who receive viagabatrin therapy. These data suggest that the debilitating effects of ABAT deficiency are a result of disturbance to ABAT function during embryonic development.

This patient illustrates how a personalized medicine approach consisting of functional vetting of an individual patient’s mutations can aid in the diagnosis of genetic disorders. This includes neurological disorders and others that commonly require extensive and invasive testing. Importantly, this patient marks an expansion in the clinical phenotype for ABAT deficiency to a milder presentation that is more commonly seen in pediatric genetics and neurology clinics.

## Abbreviations

ABAT: 4-aminobutyric acid transaminase
CSF: cerebral spinal fluid
GABA: gamma-aminobutyric acid
GABA-T: gamma-aminobutyric acid transaminase
PLICs: posterior limbs of the internal capsules
VUS: variants of uncertain significance
